# Cyclin-dependent kinase 19 upregulation correlates with an unfavorable prognosis in hepatocellular carcinoma

**DOI:** 10.1186/s12876-021-01962-8

**Published:** 2021-10-14

**Authors:** Xiaopeng Cai, Jingwen Deng, Jiaming zhou, Huiqiang Cai, Zhi Chen

**Affiliations:** 1 State Key Laboratory for Diagnosis and Treatment of Infectious Diseases, National Clinical Research Center for Infectious Diseases, Collaborative Innovation Center for Diagnosis and Treatment of Infectious Diseases, The First Affiliated Hospital, Zhejiang University School of Medicine, Hangzhou, 310003 China; 2 Department of Pathology, Key Laboratory of Disease Proteomics of Zhejiang Province, Zhejiang University School of Medicine, Hangzhou, 310058 China; 3 Women’s Hospital, Zhejiang University School of Medicine, Hangzhou, 310058 China; 4 Department of Clinical Medicine, University of Aarhus, Palle Juul-Jensens Boulevard 82, 8200 Aarhus N, Denmark

**Keywords:** Cyclin-dependent kinase 19, Hepatocellular carcinoma, Prognosis, Knockdown, Proliferation, Migration, Invasion

## Abstract

**Objectives:**

Cyclin-dependent kinase 19 (CDK19) is a component of the mediator coactivator complex, which is required for transcriptional activation. In this study, we utilized public databases and wet-bench hepatic cell line experiments to elucidate the potential roles of CDK19 in hepatocellular cancer (HCC).

**Materials and methods:**

We studied the relationships between CDK19 expression and several clinical features related to HCC via the Oncomine and UALCAN databases. The prognostic value of CDK19 was tested using the Kaplan–Meier Plotter database. We presented the mutations of CDK19 and addressed the relation of CDK19 expression with immune cell infiltration by means of the cBioPortal, Catalogue of Somatic Mutations in Cancer (COSMIC) and Tumor IMmune Estimation Resource (TIMER) databases. Hub genes were obtained and further analyzed using the Search Tool for the Retrieval of Interacting Genes/Proteins (STRING) database. To test the in silico findings, we knocked down CDK19 with short hairpin RNA (shRNA) technology in two hepatic cell lines and conducted several functional characterization experiments.

**Results:**

Marked CDK19 upregulation was found in HCC tissues versus normal liver tissues, and CDK19 mRNA expression had high diagnostic value in HCC patients. Subgroup analysis showed that CDK19 overexpression was associated with sex, tumor stage and TP53 mutation status. The prognostic value of CDK19 upregulation for overall survival (OS) was significant in patients with stage 2–3, stage 3–4, and grade 2 disease. One percent of the patients had CDK19 mutations, but no relationship between CDK19 mutation and prognosis was observed. CDK19 was positively correlated with the abundances of CD4 + T cells, macrophages and dendritic cells. We identified 10 genes correlated with CDK19, 8 of which presented excellent prognostic value in HCC. These hub genes were directly involved in cell division and regulation of the G2/M cell cycle transition. Protein–protein interaction (PPI) and pathway predictions indicated that CDK19 is highly likely to be involved in several cellular functions, such as proliferation, migration, and invasion. These functions were strongly interfered from two independent hepatic cell lines after CDK19 knockdown.

**Conclusions:**

CDK19 could be a prognostic marker in HCC, and its therapeutic potential in HCC needs further study.

**Supplementary Information:**

The online version contains supplementary material available at 10.1186/s12876-021-01962-8.

## Background

Hepatocellular carcinoma (HCC), accounting for 75–85% of primary liver cancers, ranks as the 6th most commonly diagnosed cancer and the 3rd most common cause of cancer-related death globally [1]. In recent years, the incidence of HCC has increased dramatically and will continue to rise over the next 10–20 years [2]. The major challenges are metastasis and recurrence after resection, which contribute to the dismal prognosis of HCC. Currently, several novel therapeutic options for HCC are emerging and have been shown to improve survival rates, but the overall prognosis is still unsatisfactory [3]. Thus, identifying promising prognostic biomarkers is still urgent and necessary.

Cyclin-dependent kinase 19 (CDK19) is a cyclin-dependent transcription-regulating kinase. CDK19 and its homolog CDK8, termed ‘mediator kinase’, have been shown to have crucial roles in cellular homeostasis and developmental programming. In a mutually exclusive manner, CDK19 or CDK8 can form a mediator coactivator complex with three other proteins, CCNC, MED12 and MED13 [4]. CDK19 or CDK8 reversibly regulates RNA polymerase II to control transcriptional activity. CDK8 has been reported to be involved in the development of malignancies, including cancers of the colon, breast and pancreas [5–7]. In contrast, the role of CDK19 in carcinogenesis is rarely studied and only sporadically reported in prostate cancer, colorectal cancer, breast cancer, etc. [8–10]. Moreover, some small-molecule CDK8/19 inhibitors have been found to possess beneficial effects on tumor treatment, and a clinical trial (ClinicalTrials.gov Identifier: NCT03065010) with estrogen receptor-positive breast cancer has been on its way [11]. A study found that CDK8/CycC is a further target of sorafenib, which extends into the deep pocket of the kinase [12]. Here, we performed bioinformatics analysis of HCC patients treated with sorafenib and found that they had a better prognosis than those not treated with sorafenib (Additional file 1: Fig. S1).

In this study, we first investigated the expression of CDK19 and the prognostic value of CDK19 in HCC patients. Next, we evaluated the relationship between CDK19 and immune infiltrates, identified 10 hub genes strongly correlated with CDK19, and explored the underlying roles of CDK19 by protein–protein interaction (PPI) and pathway analysis. Then, we conducted CDK19 knockdown in two HCC cell lines and confirmed its relevant functions from in vitro assays.

## Materials and methods

### mRNA/protein expression and survival analysis

We searched ‘CDK19’ as the gene symbol in the Oncomine database to explore the expression of CDK19 in HCC (http://www.oncomine.org; accessed from 2 February, 2021). CDK19 expression values from four Gene Expression Omnibus (GEO) datasets (including Roessler liver, Wurmbach liver, Roessler liver2 and Chen liver) were obtained, and dot plots were generated using GraphPad Prism 7.0 software. Then, we collected and collated the results regarding CDK19 expression levels in different subgroups using the UALCAN database (http://ualcan.path.uab.edu; accessed from 2 February, 2021), whose data were based on The Cancer Genome Atlas (TCGA) [13]. Moreover, we investigated the protein expression of CDK19 in the Human Protein Atlas database (www.proteinatlas.org; accessed from 2 February, 2021) [14, 15]. Next, CDK19 expression and overall survival (OS) in HCC patients were evaluated using the Kaplan–Meier Plotter database [16], where patients were split by the automatically selected best cutoff (http://kmplot.com; accessed from 2 February, 2021).

### Mutant and immune infiltrates analysis

We evaluated the mutation frequency of CDK19 in HCC patients using the cBioPortal database (http://www.cbioportal.org/; accessed from 2 February, 2021) [17, 18]. We selected all 1,000 listed HCC samples from 5 studies as study objects. Then, we validated the mutation of CDK19 in HCC in the Catalogue of Somatic Mutations in Cancer (COSMIC) database (http://cancer.sanger.ac.uk; accessed from 2 February, 2021) [19, 20]. Next, we further explored the associations between CDK19 and immune cells using the Tumor Immune Estimation Resource (TIMER) database (https://cistrome.shinyapps.io/timer/; accessed from 2 February, 2021) [21].

### Differential expression genes and hub genes analysis

We investigated the differentially expressed genes (DEGs) using the LinkedOmics database, which contains multiomics data for 32 cancer types (http://www.linkedomics.org; accessed from 2 February, 2021) [22]. A total of 371 TCGA-HCC RNA-seq samples were analyzed by Spearman’s test. Then, the hub genes were determined using Cytoscape software based on the top 200 significantly correlated DEGs (https://cytoscape.org/; accessed from 2 February, 2021). The prognostic significance of hub genes and the association between CDK19 and hub genes were investigated using the Kaplan‐Meier Plotter database [16] and Gene Expression Profiling Interactive Analysis (GEPIA) (http://gepia.cancer-pku.cn/; accessed from 2 February, 2021) [23].

### Protein–protein interaction network and GO/KEGG analysis

PPI analysis for CDK19 was performed using the Search Tool for the Retrieval of Interacting Genes/Proteins (STRING) database (https://string-db.org/; accessed from 2 February, 2021) [24]. We selected the top 200 significantly related genes to establish the cluster of the network with the default criteria. Next, we conducted Kyoto Encyclopedia of Genes and Genomes (KEGG) pathway and Gene Ontology (GO) biological process enrichment analyses, and the results were visualized with the bioinformatics online tool (http://www.bioinformatics.com.cn; accessed from 2 February, 2021).

### Reagents and cell culture

HCC cell lines (Hep.G2 and Huh7) were purchased from the Chinese Academy of Sciences. Both cell lines were certified and confirmed to be free of mycoplasma infection. These cells were cultured in DMEM with 10% fetal bovine serum and maintained in an environment suitable for cell growth.

### Stable cell lines

To get stable CDK19 knockdown cell lines, we used lentivirus based short hairpin RNA (shRNA) deliver system (purchased from Genomeditech, Shanghai, China), which targets the sequence 5′-GCTTGTAGAGAGATTGCACTT-3′ (CDK19-sh1) and 5′-GCATGACTTGTGGCATATTAT-3′ (CDK19-sh2) (Gene ID: 23,097). Another lentivirus containing a scrambled shRNA (5′-TTCTCCGAACGTGTCACGT-3′) was employed as the non-targeting control of our method. After 48 h of lentiviral transduction, we transferred cells into 1 μg/mL puromycin medium for 10 days to select knockdown cells. Then, the mRNA expression of CDK19 was evaluated. Total cellular RNA was extracted, and relative mRNA expression was determined. The primers for CDK19 were as follows: forward primer 5ʹ-GTTTCACCGTGCATCAAAAGC-3ʹ; reverse primer 5ʹ-ACCCAATTTGCATGGAGGTAATG-3ʹ. GAPDH was set as an internal reference gene.

### Cell proliferation assay

Cells transfected with CDK19-SC or sh-CDK19 were seeded evenly into 96‐well plates at 2,000 cells/well. Then, we detected cell viability using Cell Counting Kit-8 (CCK-8) at 0 h, 24 h, 48 h, 72 h and 96 h. These cells were incubated with 10 µl CCK-8 for 2 h, and the absorbance at 450 nm was recorded using a VICTOR Nivo Multimode Plate Reader (PerkinElmer Inc., Massachusetts, USA).

### Migration and invasion assay

Transwell migration and transwell invasion assays were performed to evaluate the migration and invasion ability of cells. Briefly, cell suspension of Hep.G2 and Huh7 were plated into the upper chamber (Corning Falcon, USA) with serum-free medium and the medium of lower chamber was containing 10% FBS. Diluted extracellular matrix gel (BD, MA, USA) was added to the upper chamber for the invasion assays, but not migration assays. 2 × 10^5^ cells of Hep.G2 and 1 × 10^5^ cells of Huh 7 were used for migration and invasion assays. After incubated for 24 h (Huh7) or 72 h (Hep.G2), the cells in the upper chamber were fixed with 4% paraformaldehyde and stained by crystal violet (Beyotime, China). The photos were taken to count the number of migration and invasion cells.

### Statistical analysis

We used GraphPad Prism 7.0 software to analyze some of the results. The data were summarized as the mean ± SEM. Differences between 2 groups were evaluated using Student’s t-test.

## Results

### High expression of CDK19 in HCC

**Fig. 1:**
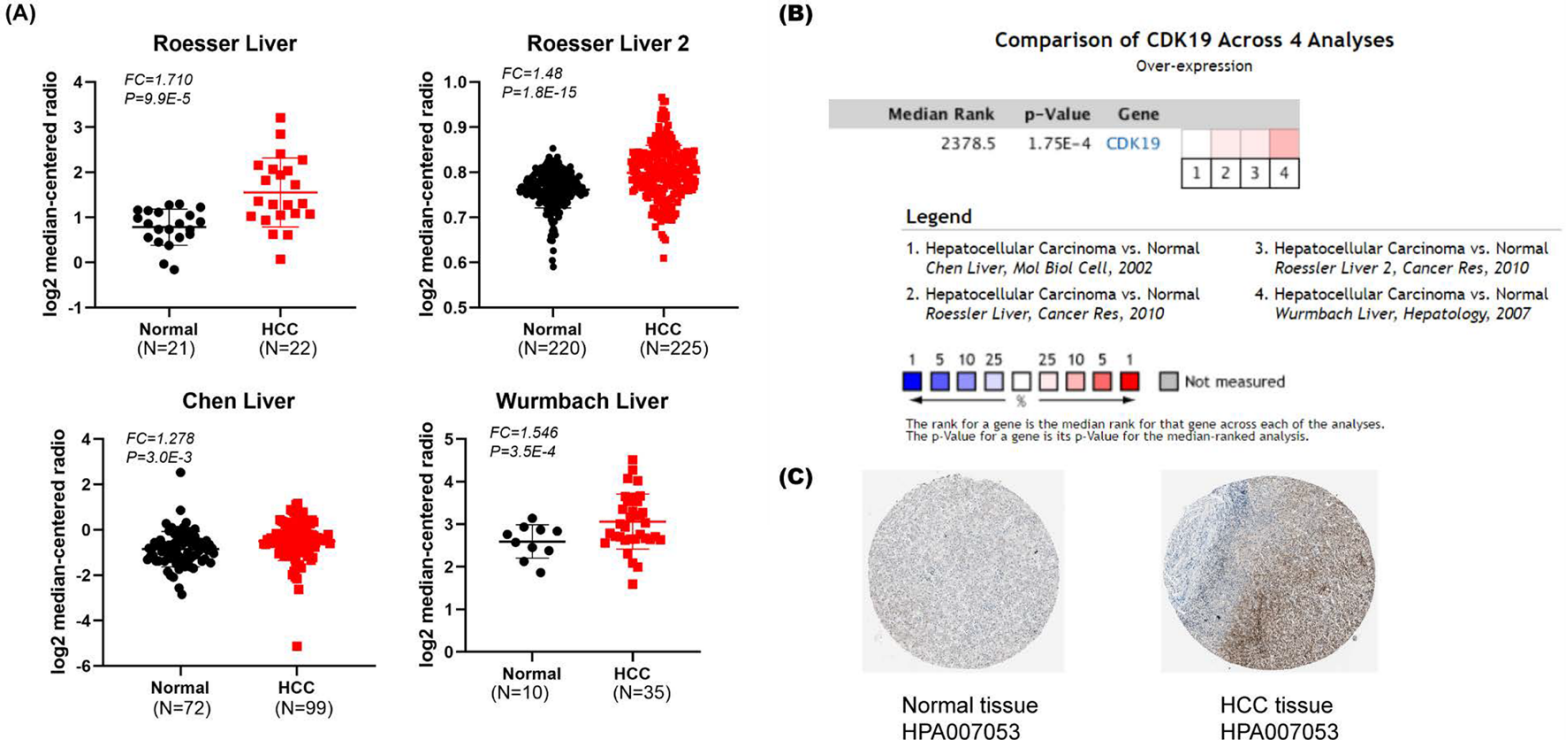
The overexpression of CDK19 in hepatocellular carcinoma (HCC). **A** CDK19 is overexpressed in several types of cancers (overexpression-red colour, downexpression-blue colour). **B** CDK19 is overexpressed in HCC. **C** Protein expression of CDK19 is elevated in HCC tissues

CDK19 is a cyclin-dependent transcription-regulating kinase. Both CDK19 and its homolog CDK8, termed mediator kinase, have been shown to play crucial roles in cellular homeostasis and to be related to several diseases. Based on Oncomine, we evaluated the expression of CDK19 in HCC from 4 GEO datasets (Roessler liver, Roessler liver 2, Chen liver, and Wurmbach liver) [25–27]. As a result, the expression of CDK19 in HCC tissues was significantly upregulated. For example, CDK19 showed a 1.71-fold increase in the HCC versus normal tissues in the Roessler liver datasets (Fig. 1A). The difference in CDK19 expression across these four studies was significant, indicating that interpatient variations may exist to some extent. (*P* < 0.05) (Fig. 1B). We next investigated the protein expression of CDK19 in HCC by using the Human Protein Atlas (HPA) database. As shown in Fig. 1C, CDK19 was hardly detected in normal liver tissue (Patient ID 2556), but from liver tumor tissue (Patient ID 2177), it showed a strong signal (Fig. 1C).

**Fig. 2:**
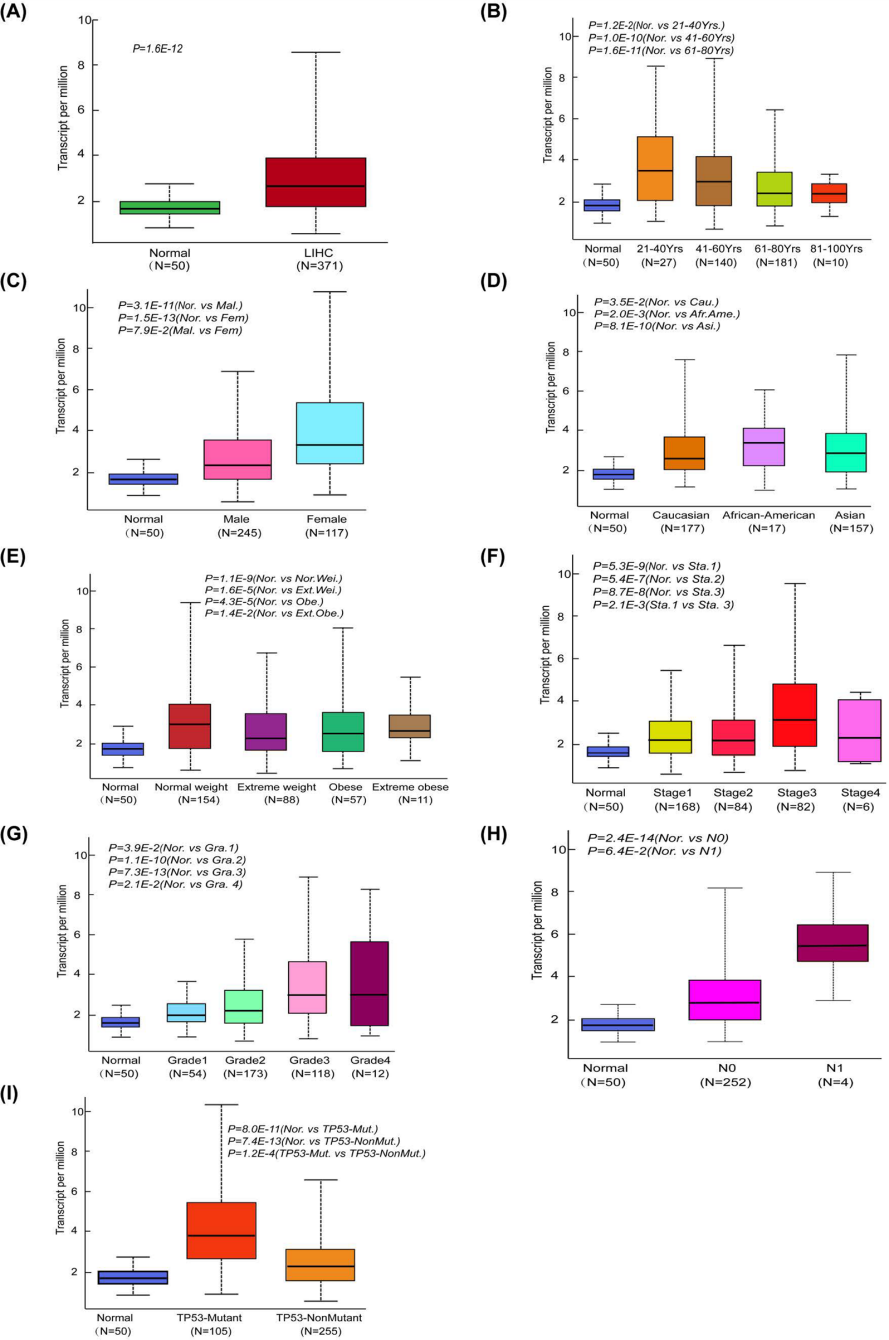
Subgroups expression analysis of CDK19 in HCC using UALCAN database. **A** mRNA expression of CDK19 in normal tissue and LIHC tissues. **B**–**E** CDK19 mRNA expression levels of HCC patients in subgroups with different ages, genders, races and weights. **F**–**H** CDK19 mRNA expression levels of HCC patients with different tumour stages, tumour grades and metastasis status. **I** CDK19 mRNA expression levels of HCC patients with TP‐53 mutant

To further explore the interpatient variations in CDK19, we studied the expression patterns of CDK19 in the TCGA-LIHC cohort by using UALCAN and considered several different clinical features. As shown in Fig. 2A, CDK19 had much higher expression in LIHC patients (n = 371) than in the normal control group (n = 50) (Fig. 2A). While clustering the patients into different subgroups based on their age, sex, race and weight, we observed differential expression profiles (Fig. 2B–E). For example, the expression difference was not significant between the control group and patients at the age of 81–100 years. In addition, there seemed to be a correlation between CDK19 expression and the severity of different tumors (Fig. 2F–H). For example, the difference in expression between stage 1 and stage 3 patients was quite obvious (*P* = 0.0021). Considering that TP53 is one of the most commonly mutated genes in HCC, we found that there may be a correlation between TP53 and CDK19. Intriguingly, CDK19 expression was much higher in patients with TP53 mutations than in those without TP53 mutations (*P* = 0.00012) (Fig. 2I).

### Survival results and multivariate analysis in HCC

**Fig. 3:**
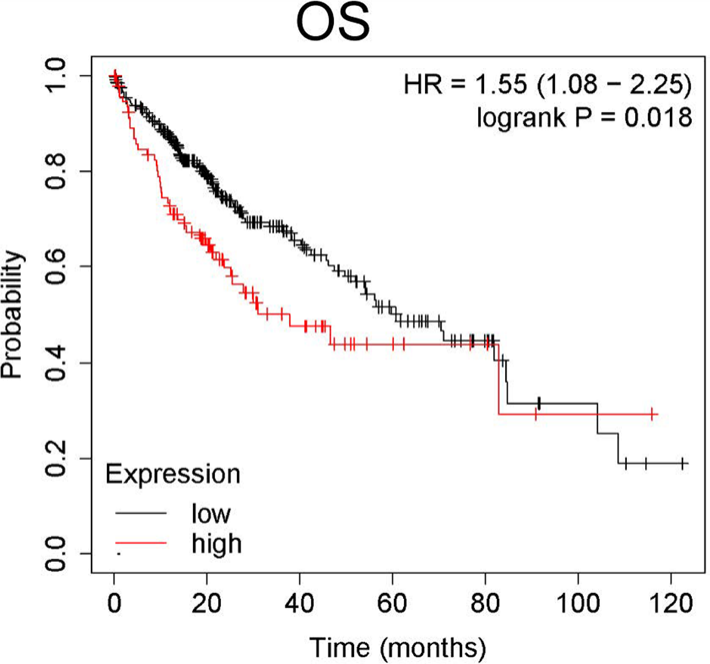
The overall survival (OS) values were analyzed in regards to the mRNA expression level of CDK19 in all HCC patients

We evaluated the prognostic significance of CDK19 in 364 patients using the Kaplan‐Meier Plotter database. We found that the higher the expression of CDK19 was, the worse the OS (HR = 1.55, log‐rank *P* = 1.8E−2) of HCC patients was (Fig. 3). The prognostic value of CDK19 in different clinical subgroups of HCC was investigated. The results indicated that OS was relatively poor in patients with high CDK19 mRNA expression under the conditions of stage 2–3, stage 3–4, or grade 2 disease; male sex; Asian race; alcohol consumption and non-hepatitis virus infection (Additional file 1: Fig. S1). Collectively, these data indicate that the CDK19 expression level can serve as a valuable prognostic biomarker in HCC patients and that the prognostic significance varies depending on different clinical subgroups, which can guide our clinical practice in a personized manner.

### Mutations of CDK19 in HCC

**Fig. 4:**
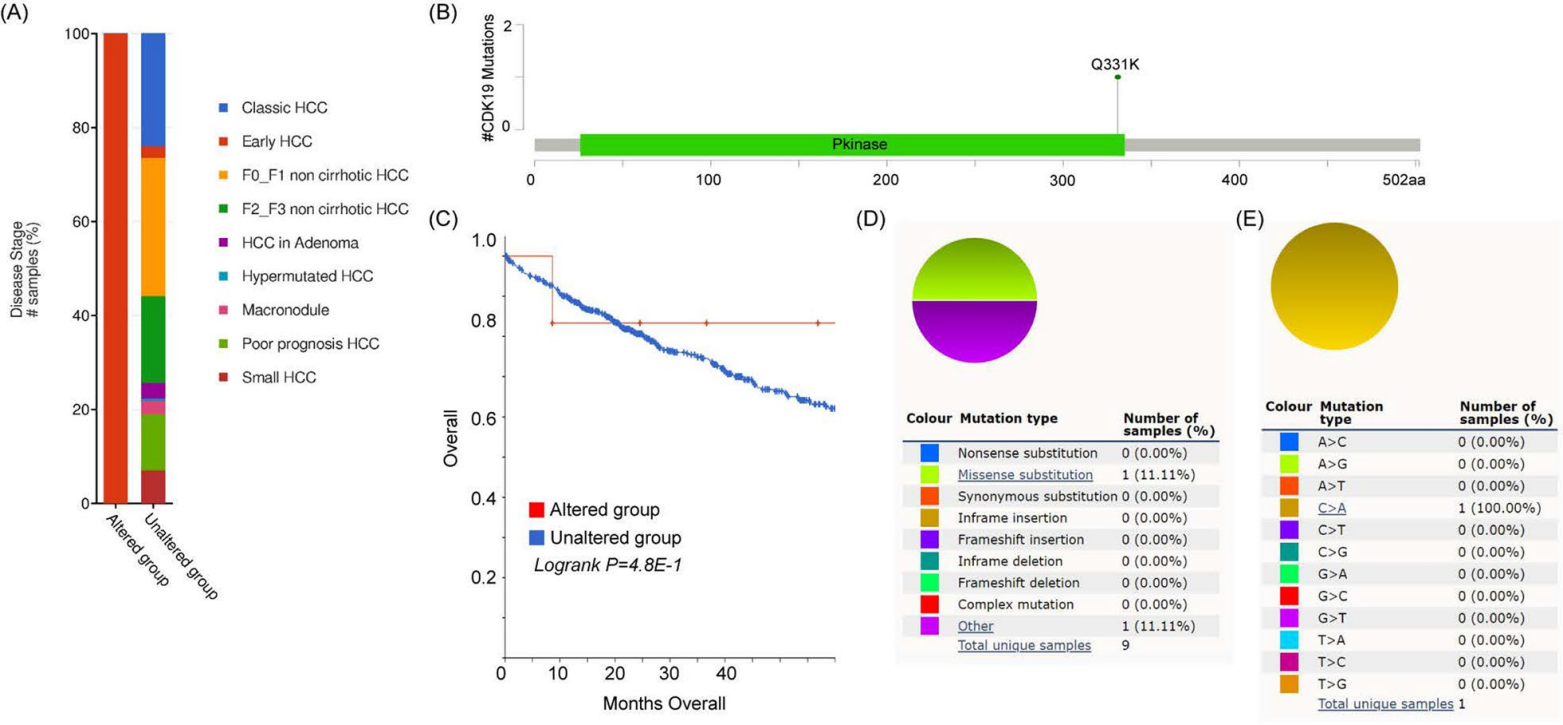
CDK19 mutations in hepatocellular carcinoma (HCC). **A** The alterations of CDK19 gene in HCC. **B** The schematic representation of CDK19 mutations in HCC. **C** The relationship between CDK19 alterations and the prognosis of HCC patients. **D**–**E** The mutation types of CDK19 (%) in HCC

Next, we investigated the mutation landscape of CDK19 in a large number of HCC patients by using cBioPortal software. Overall, 1,000 samples from 998 patients allocated in five studies (AMC, INSERM, RIKEN, MSK and TCGA‐PanCancer Atlas) were selected for analysis [28–32]. From our data mining, 8 alterations of CDK19 were found, with one missense mutation that appeared in early HCC (Fig. 4A). The somatic mutant frequency of CDK19 was approximately 1% (Fig. 4B). However, CDK19 somatic status could not be used to distinguish OS in HCC patients (Fig. 4C). In addition, we used the COSMIC database to verify the mutation of CDK19 in HCC. There were only 9 mutations from 951 tissues (somatic mutation frequency: 0.95%), and the only type of mutation was missense substitution (Fig. 4D). The substitution mutations were all C > A changes (100%) (Fig. 4E).

### The relationship between CDK19 and immune infiltrates in HCC

**Fig. 5:**
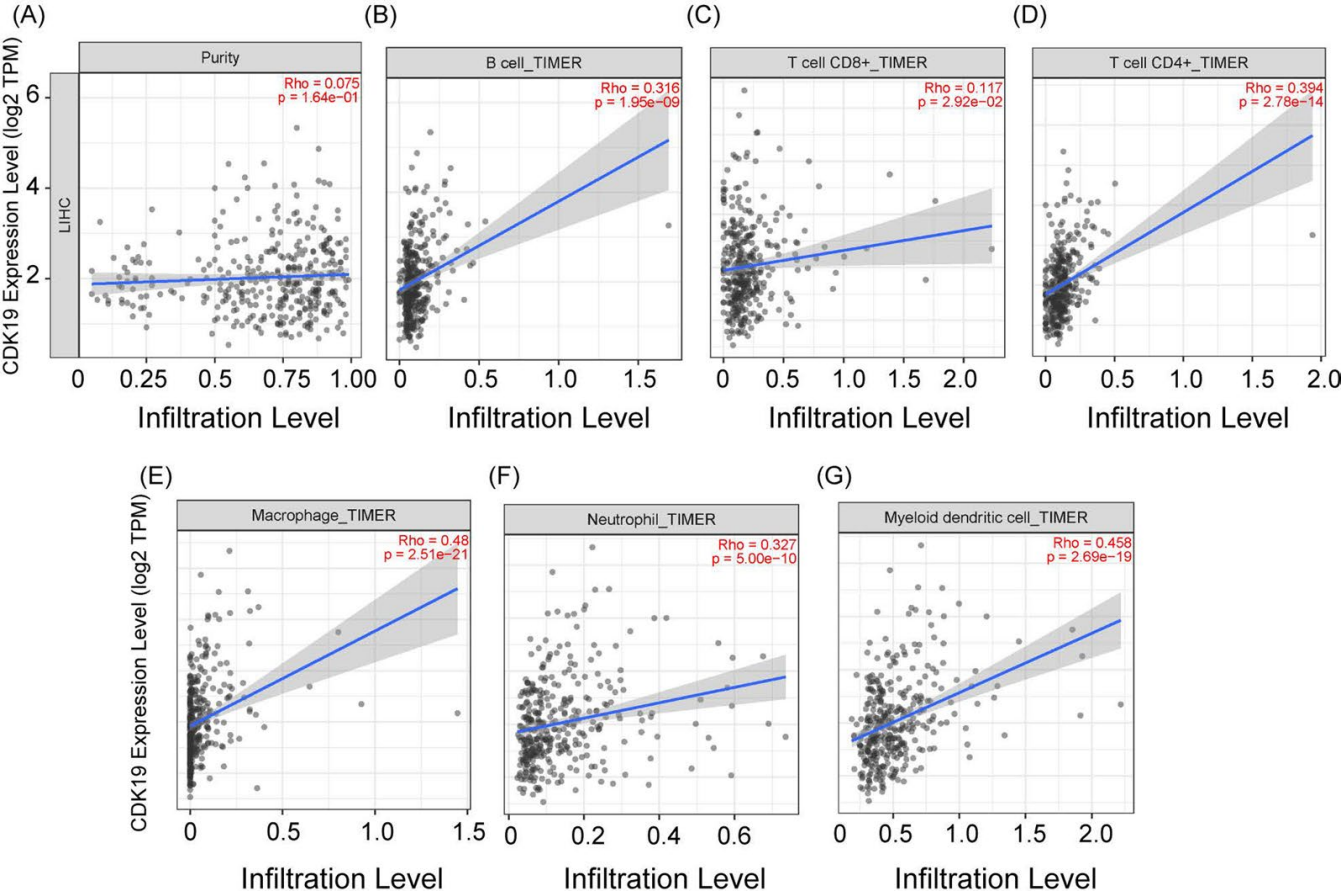
CDK19 was associated with immune infiltration in hepatocellular carcinoma (HCC). **A** The correlation between CDK19 and tumor purity. **B**–**G** Graphs showed the correlations between CDK19 and **B** B cell, **C** T cells CD8 + , **D** T cells CD4 + , **E** macrophage, **F** neutrophil and **G** myeloid dendritic cell

To investigate the correlation between CDK19 and immune infiltrates, we used the TIMER online tool. The relationships between 6 immune cell types (B cells, CD8 + T cells, CD4 + T cells, macrophages, neutrophils and myeloid dendritic cells) and CDK19 expression were determined by Spearman tests (tumor purity adjusted, Fig. 5A). CDK19 was significantly positively correlated with these 6 immune infiltrates, especially macrophages (R = 0.48) and myeloid dendritic cells (R = 0.458) (Fig. 5B–G). This inspired us to further research whether there may be a potential association between CDK19 and immune cell gene markers. Similar to the findings above, CDK19 had positive correlations with the respective gene markers of those 6 immune cells (Table 1). Among the listed gene markers, QRSL1 (R = 0.700), IRF5 (R = 0.483), STAT1 (R = 0.469), NRP1 (R = 0.469) and PTGS2 (R = 0.467) were the most relevant (Table 1).

**Table 1:** Correlations between CDK19 and immune cells’ gene markers in HCC

Cells subtypes	Markers	Non-purity adjusted		Purity adjusted	
		ρ(rho)	*P*	ρ(rho)	*P*
B cells	CD19	0.213	4.90E−04	0.245	8.60E−05
	CD79A	0.189	2.60E−03	0.26	1.90E−05
T cells (general)	CD3D	0.173	4.70E−03	0.237	1.80E−04
	CD2	0.183	2.30E−03	0.272	5.80E−06
	CD3E	0.182	2.10E−03	0.278	3.20E−06
CD8 + T cells	CD8B	0.126	5.00E−02	0.186	2.60E−03
	CD8A	0.195	1.20E−03	0.265	7.70E−06
CD4 + T cells	QRSL1	0.698	2.60E−54	0.7	5.10E−51
	STAT1	0.443	4.20E−18	0.469	5.30E−19
	STAT4	0.312	1.60E−08	0.348	3.70E−10
	STAT5A	0.404	7.60E−15	0.435	3.10E−16
	STAT6	0.339	1.10E−10	0.335	1.00E−09
	CD4	0.303	1.70E−08	0.362	5.20E−11
	TBX21	0.096	1.60E−01	0.16	9.40E−03
Tumur associated macrophages	CCL2	0.217	2.10E−04	0.289	4.50E−07
	CD68	0.193	1.30E−03	0.239	6.90E−05
	IL10	0.264	1.00E−05	0.34	1.80E−09
Type I macrophages	IRF5	0.483	1.90E−21	0.483	6.40E−20
	NOS2	0.156	6.10E−03	0.172	3.30E−03
	PTGS2	0.364	8.50E−12	0.467	1.60E−18
Type II macrophages	CD163	0.15	1.00E−02	0.216	1.70E−04
	MS4A4A	0.156	8.60E−03	0.235	5.40E−05
	VSIG4	0.173	3.40E−03	0.245	2.90E−05
Neutrophil	CCR7	0.181	2.30E−03	0.26	9.90E−06
	ITGAM	0.315	5.20E−09	0.365	2.60E−11
Dendritic cells	CD1C	0.252	6.00E−06	0.3	1.30E−07
	HLA-DPB1	0.175	3.70E−03	0.234	7.30E−05
	HLA-DQB1	0.086	2.80E−01	0.133	4.80E−02
	HLA-DRA	0.171	4.60E−03	0.23	7.90E−05
	ITGAX	0.337	1.00E−09	0.425	6.20E−15
	NRP1	0.459	1.00E−19	0.469	2.70E−19

### The genes correlated with CDK19 in HCC

**Fig. 6:**
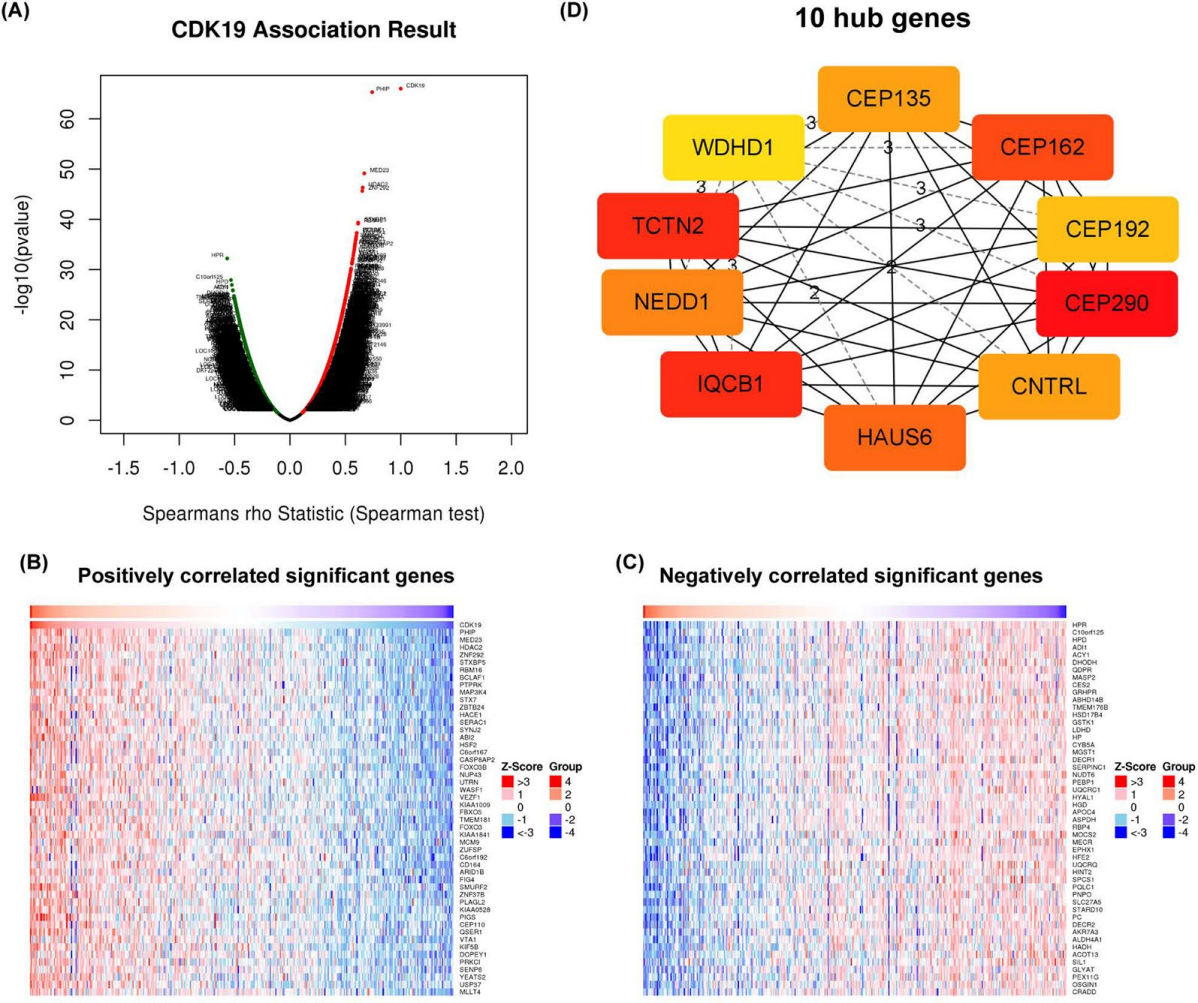
Genes correlated with CDK19 in hepatocellular carcinoma (HCC). **A** Correlations between CDK19 and differently expressed genes (DEGs). **B**, **C** The positively or negatively correlated genes with CDK19 (Top 50 genes). **D**The 10 hub genes of CDK19 in HCC

We investigated the genes correlated with CDK19 using LinkedOmics software. As the volcano map shows (Fig. 6A), the negatively and positively related genes were located in the left and right areas, respectively. The top 50 positively and negatively related genes were identified based on the Spearman test and are shown in heatmaps separately (Fig. 6B–C). To address whether some hub genes existed, we input the top 200 positively related genes with CDK19 into the STRING online database and Cytoscape software. Based on gene degree, the 10 most relevant hub genes, including CEP135, CEP162, CEP192, CEP290, CNTRL, HAUS6, IQCB1, NEDD1, TCTN2 and WDHD1, were obtained (Fig. 6D). We were surprised to find that almost all the hub genes are directly involved in cell division and regulation of the G2/M transition of the mitotic cell cycle. The correlations between CDK19 and the 10 hub genes were validated by using the GEPIA web tool (Additional file 2: Fig. S2). Finally, we found that 8 of the 10 top hub genes presented excellent prognostic value in HCC (Additional file 3: Fig. S3), especially IQCB1 (HR = 2.05) and NEDD1 (HR = 1.93) (Fig. 6E).

### PPI and KEGG/GO enrichment of CDK19 in HCC

**Fig. 7:**
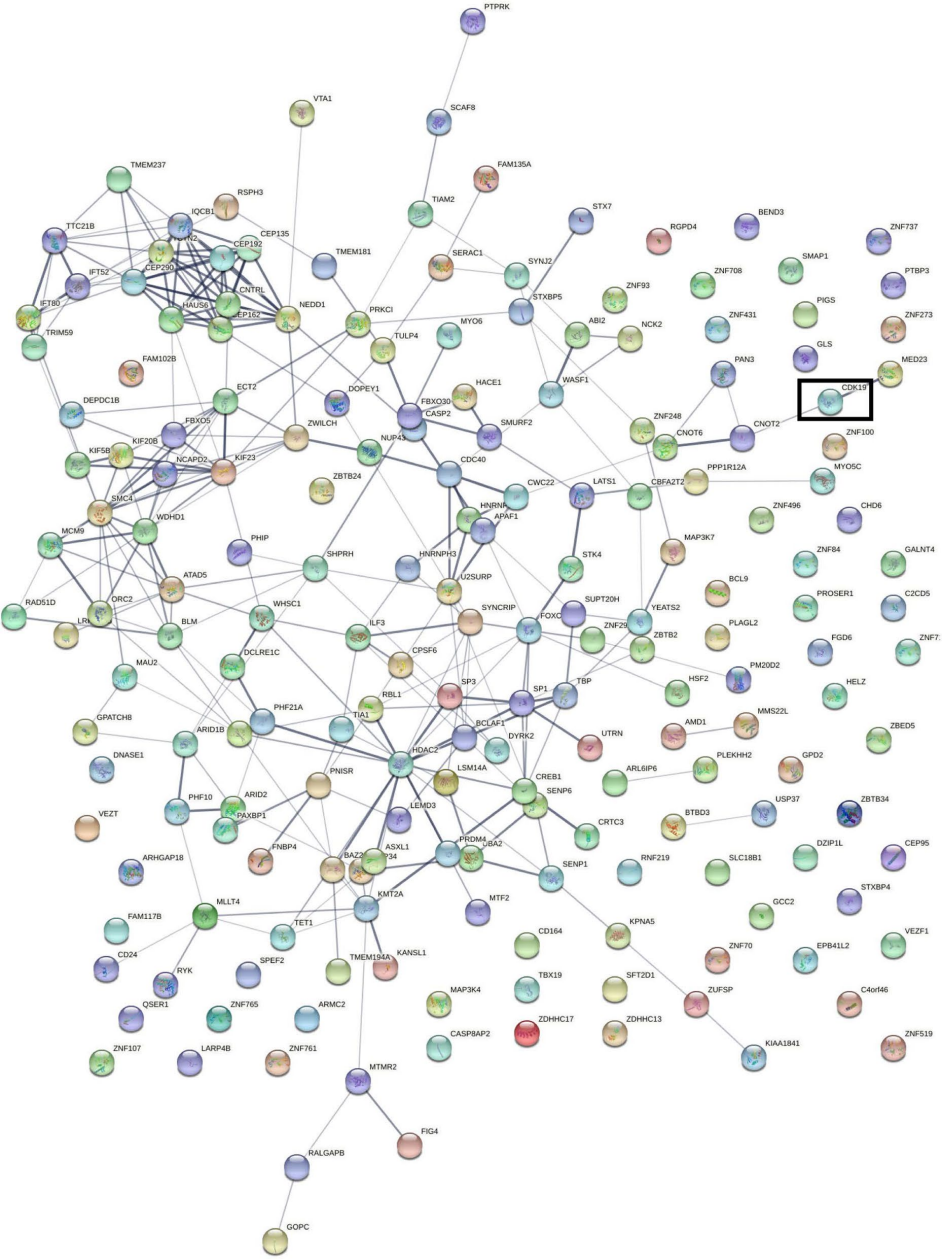
The Protein–Protein Interaction (PPI) network of the top 200 genes in hepatocellular carcinoma (HCC)

By utilizing STRING software, we constructed a PPI network based on the top 200 significantly related genes (Fig. 7). As shown in the network, CDK19 can directly interact with MED23 and CNOT2, through which it further interacts with other proteins. MED23 and CNOT2 are both involved in the regulation of gene expression and transcription. We also found that many proteins in the network took part in tumor development; for example, PHIP drives glioblastoma motility and invasion [33], HDAC2 regulates breast cancer progression and proliferation, [34] and ZFN292 participates in hepatoma proliferation and vasculogenic mimicry [35].

**Fig. 8:**
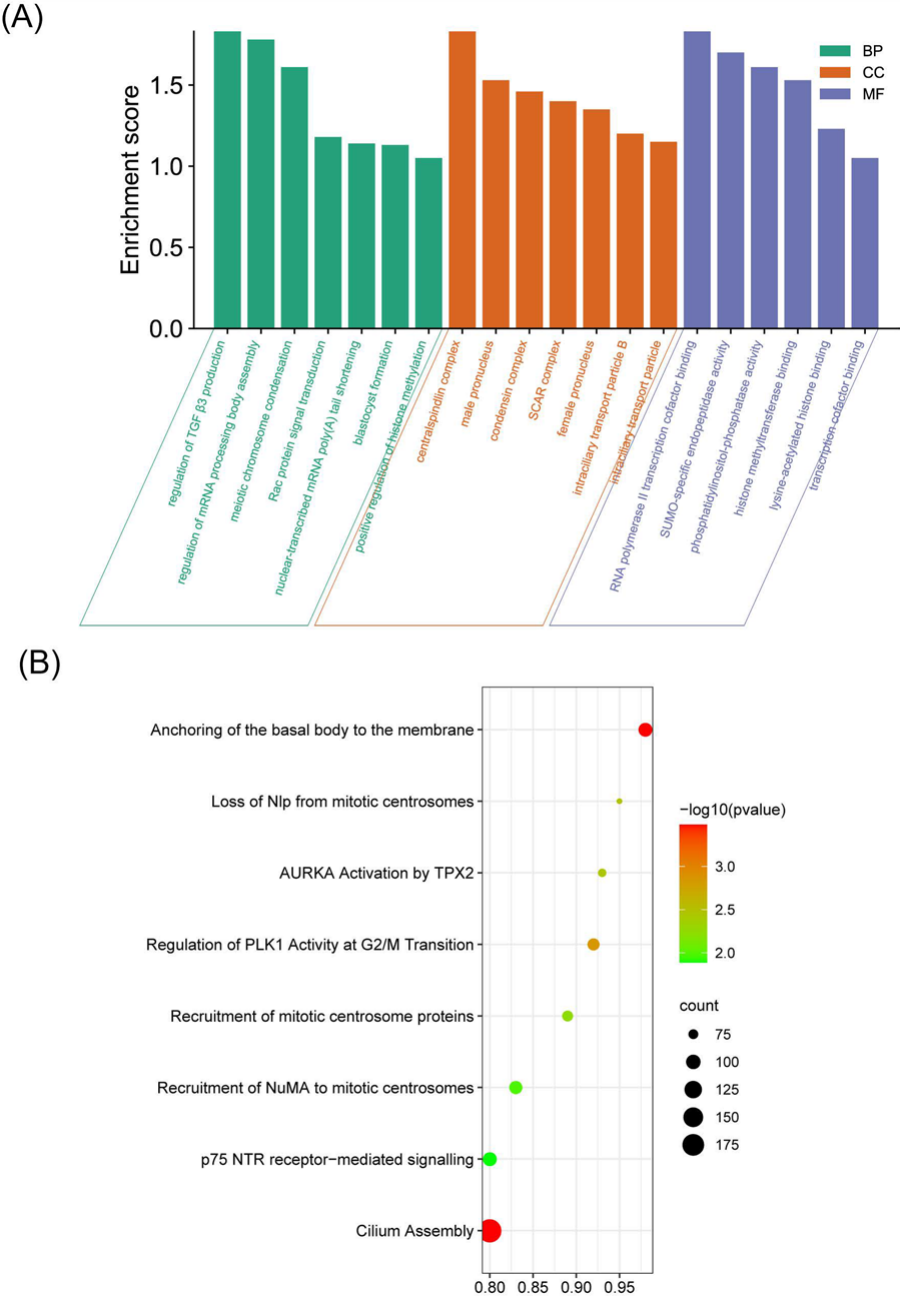
The Kyoto Encyclopedia of Genes and Genomes (KEGG) pathway and Gene Ontology (GO) biological process analysis of CDK19 in hepatocellular carcinoma (HCC). **A**The GO biological process analysis of CDK19 in HCC. **B**The KEGG pathway enrichment analysis of CDK19 in HCC

Then, we investigated the enriched GO/KEGG processes and signaling pathways in which CDK19 may be involved. GO biological process analysis of CDK19 showed that binding to transcription cofactors, regulation of transcription factors and mRNA were significantly affected and enriched (Fig. 8A). KEGG pathway enrichment showed that CDK19 is mainly involved in mitosis in different ways (Fig. 8B).

### CDK19 is involved in several cellular functions of hepatic tumor cell lines

**Fig. 9:**
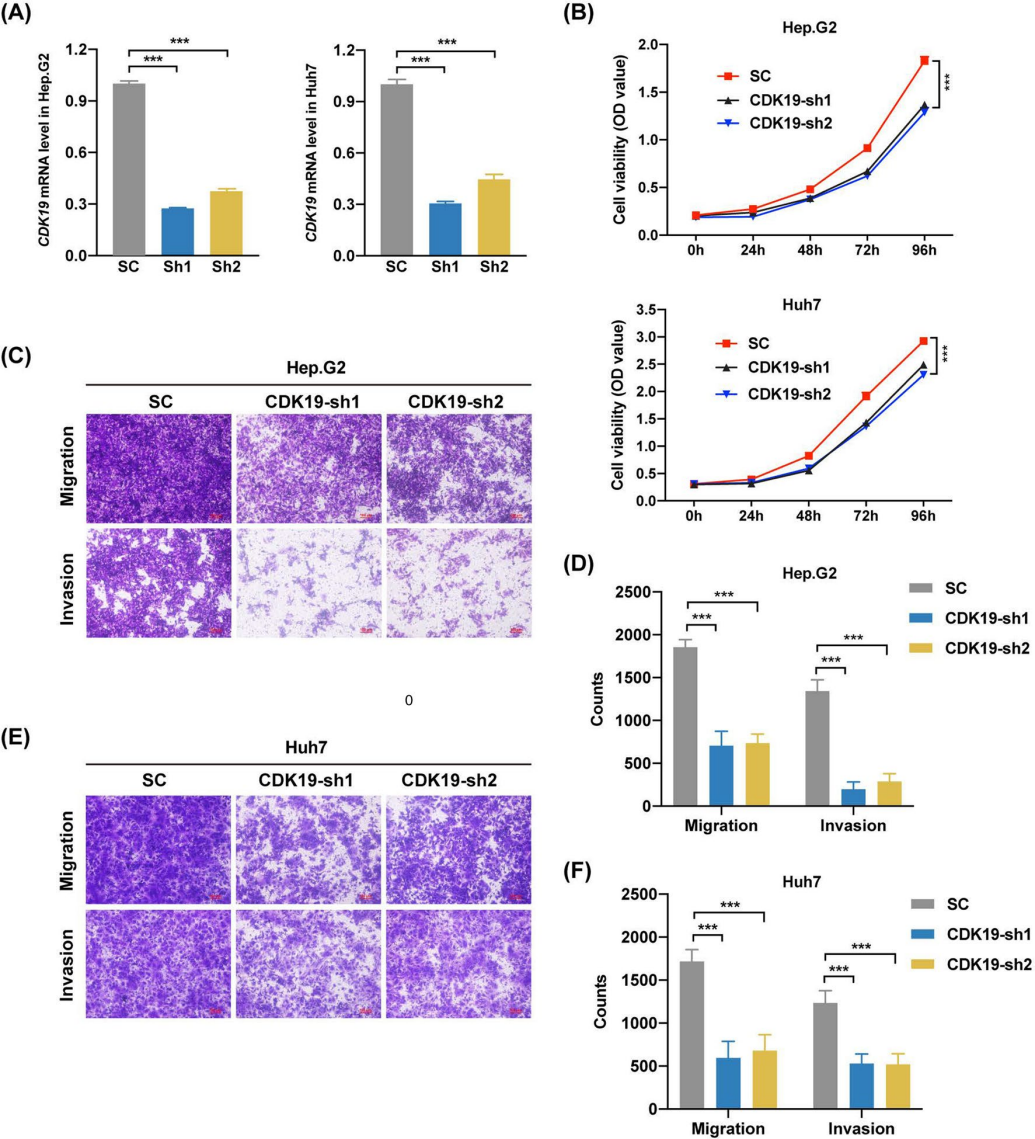
Functional analysis of CDK19 in hepatocellular carcinoma (HCC) cells. **A** mRNA expression of CDK19 after CDK19-SC or sh-CDK19 transfection. **B** Cell viability measured by cell counting kit-8 at 0 h, 24 h, 48 h, 72 h and 96 h after CDK19-SC or sh-CDK19 transfection. The migration and invasion assays of Hep.G2 (**C**) and Huh7 (**D**) after CDK19-SC or sh-CDK19 transfection. *P < .05; **P < .01; ***P < .001

To validate the findings from bioinformatics analysis, we chose two independent hepatic cell lines, Hep.G2 and Huh7, as our in vitro models. Here, a lentivirus based short hairpin RNA (shRNA) delivery system was utilized to knock down CDK19. After lentiviral transduction, we selected knockdown cells with puromycin, isolated mRNA from the cells and then performed qPCR to check the CDK19 level. As shown in Fig. 9A, CDK19 was knocked down successfully in both cell lines, by comparing to a non-targeting scramble control (SC). To address whether CDK19 is involved in cell growth, we next conducted a cell viability assay. In comparison to the control, knockdown of CDK19 clearly inhibited the proliferation of Hep.G2 and Huh7 cells (Fig. 9B).

As suggested by the bioinformatics analysis, CDK19 may be directly or indirectly related to migration and invasion abilities through interaction with other invasion-relevant proteins. To validate these abilities, transwell migration and transwell invasion assays were performed in the two cell lines. As shown in Fig. 9C, E, much fewer tumor cells migrated in the knockdown group than in the control group. To confirm that CDK19 contributes to invasion ability, we performed a transwell invasion assay in the two cell lines. Similar to the above results, knockdown of CDK19 significantly decreased the number of invaded tumor cells.

Taken together, these findings indicate that knockdown of CDK19 can decrease the proliferation, migration and invasion abilities of hepatic tumor cells, suggesting that CDK19 may serve as a promising therapeutic target in HCC.

## Discussion

HCC is one of the most aggressive cancers and has a poor prognosis [36]. Globally, HCC accounts for approximately 750,000 new cases and 780,000 cancer-related deaths every year [37]. Because of late diagnosis, metastasis and quick progression, HCC has become a major killer among cancers. Currently, effective therapeutic strategies for HCC patients are still limited. However, with the development of next-generation sequencing and multiomics, the cellular and molecular mechanisms responsible for HCC tumorigenesis have gradually become clear [38]. Moreover, many new biomarkers for HCC have been discovered, and these findings provide valuable information for the diagnosis and/or prognosis evaluation of HCC.

The human mediator complex (MED) is a transcriptional regulator consisting of 30 subunits. It has 4 distinct MED modules, including the head, middle, tail and kinase modules. By interacting with RNA‐polymerase‐II and transcription factors, MED can affect almost all cellular processes, such as elongation, initiation, and chromatin architecture [39]. It is not surprising that genetic variations or changes in MED subunits contributes to the pathogenesis of many diseases, including cancers. For example, the CDK8 kinase module and MED29 promoted the expression of β-catenin in colorectal cancer, and they were shown to have oncogenic and tumor-suppressive functions, respectively, in pancreatic cancer cells [6, 40]. Moreover, a few studies found that MED1/17 and MED19 were extremely expressed in prostate cancer and breast cancer. By reducing the expression of MED1/17 and MED19, the growth and proliferation of cancer cells were inhibited [41, 42]. Therefore, a particular mediator subunit was proposed as a hallmark or therapeutic target in cancers. CDK19 and its gene paralog CDK8 are part of the mediator’s kinase module, which reversibly associate with the mediator core structure and regulate gene transcription activity by being signaling pathway coactivators and corepressors [43]. As previously mentioned, several studies found that CDK19 and CDK8 are involved in diverse cancer entities [5–10]. However, to our knowledge, the detailed characteristics of CDK19 in HCC are not yet well understood. Here, we tried to answer the following questions with great enthusiasm. First, does CDK19 participate in the pathogenesis of HCC? Second, what is the potential mechanism if CDK19 gets involved in HCC? Third, what mutations of CDK19 occur in HCC? Do these mutations have a relationship with prognosis? To answer such questions, we conducted a series of informatics analyses and experiments in this study.

With the help of dry-bench analysis and wet-bench experiments, we found that CDK19 is upregulated in HCC patients. Additionally, CDK19 overexpression was associated with different clinicopathological parameters, especially tumor stage, tumor grade, metastasis and TP53 mutation status. These results suggest that HCC patients with high levels of CDK19 are more prone to having tumors that are more advanced in terms of tumor stage, tumor grade, metastasis and TP53 mutation status than those with low levels of CDK19 expression. Moreover, high expression of CDK19 showed tight correlations with the clinical features of HCC patients, such as OS. This indicated that CDK19 can be used as a novel prognostic marker in HCC. Additionally, we found that OS was significant in patients with stage 2–3, stage 3–4, and grade 2 disease; male sex; Asian race; alcohol consumption; nonhepatitis infection; and sorafenib treatment. These results further proved that CDK19 can be employed to assess the prognosis of different HCC patients. Currently, immune checkpoint inhibitors (ICIs) have shown good prospects in various types of malignant tumors. In HCC patients, antibodies against programmed cell death-1 (anti-PD-1) or its ligand (anti-PD-L1) are the backbones of numerous combination regimens [44]. To explore whether there is a possibility to combine CDK19 targeting and canonical immunotherapy, we researched whether there was a potential relationship between CDK19 expression and immune infiltration in HCC. Our work showed that the expression of CDK19 was positively correlated with immune infiltration, which indicated that CDK19 may play an important role in the HCC immune microenvironment. Hofmann et al. recently found that CDK8/19 inhibitors could enhance the killer function of NK cells and promote the lysis of primary leukemia cells [45]. As mentioned above, sorafenib can target CDK18/CycC by extending into the pocket of the kinase [12]. The ICI therapeutic effect varies widely between different cancer types and different individuals because of intratumoral heterogeneity, key gene mutations, etc. [46]. Therefore, we further explored the mutation of CDK19 in HCC. We found that the mutation frequency was only 1% in HCC patients, and most mutations were missense substitutions. There were no correlations between mutation and prognosis. These works inspired us to use CDK19 inhibitors in the treatment of HCC in the future, as CDK19 is a relatively conserved gene.

With data mining for the PPI network and GO/KEGG analysis, we found that CDK19 participates in the regulation of some critical transcription factors and may be involved in mitosis, proliferation, invasion and migration at the mRNA level. For example, CDK19 involves mRNA processing body assembly and RNA polymerase II transcription cofactor binding and regulates PLK1 activity at the G2/M transition. PLK1 plays an important role in the initiation, maintenance, and completion of mitosis [47]. Hence, we further explored whether CDK19 could affect the malignancies of HCC cell lines. A series of phenotypic functional experiments demonstrated that CDK19 may participate in HCC development by promoting proliferation, migration and invasion. These phenotypic characteristics are similar to those of CDK19 in prostate cancer, gastric cancer, and head and neck squamous cell carcinoma [48–50]. Above all, CDK19 is an oncogene and plays an important role in the pathology of HCC. As an ICI target, CDK19 is worthy of further study.

## Conclusions

In conclusion, we first demonstrated that upregulated CDK19 expression was correlated with a poor prognosis in HCC based on several clinic-related features. CDK19 is positively correlated with immune infiltration. We hypothesized that CDK19 is involved in several cellular functions, such as proliferation, migration, and invasion. These findings highlight the potential value of CDK19 expression as a prognostic marker, and targeting CDK19 deserves further work to test its therapeutic use for HCC.

## Supplementary Information

**Additional file 1: Fig. 1**. The overall survival (OS) values were analyzed in regards to the mRNA expression level of CDK19 in differential subgroups of HCC patients.

**Additional file 2: Fig. 2**. The relevance of CDK19 gene expression in relation to the top 10 hub genes.

**Additional file 3: Fig. 3**. The prognostic significance of the top 10 hub genes.

## Data Availability

The datasets generated and analyzed were obtained from online databases including Oncomine (http://www.oncomine.org), UALCAN (http://ualcan.path.uab.edu), Human Protein Atlas database (www.proteinatlas.org), Kaplan‐Meier Plotter (http://kmplot.com), Catalogue of Somatic Mutations in Cancer (COSMIC) (http://cancer.sanger.ac.uk), cBioPortal (http://www.cbioportal.org), Tumor Immune Estimation Resource (TIMER) (https://cistrome.shinyapps.io/timer), LinkedOmics (http://www.linkedomics.org), cytoscope software (https://cytoscape.org), GEPIA (http://gepia.cancer-pku.cn), STRING (https://string-db.org), bioinformatics online tool (http://www.bioinformatics.com.cn).

## Abbreviations

CDK19: Cyclin dependent kinase 19
HCC: Hepatocellular cancer
DEGs: Differential expression genes
GEPIA: Gene expression profiling interactive analysis
MED: Mediator complex
COSMIC: Catalogue of Somatic Mutations in Cancer
TIMER: Tumor immune estimation resource
KEGG: Kyoto encyclopedia of genes and genomes
GO: Gene ontology
shRNA: Short hairpin RNA
