# Supplementary material for: Cyclin-dependent kinase 19 upregulation correlates with an unfavorable prognosis in hepatocellular carcinoma
Source: BMC Gastroenterol. 2021 Oct 14;21:377. doi: 10.1186/s12876-021-01962-8 (PMC8518165; doi:10.1186/s12876-021-01962-8)
Supplement: Supplementary file 1 — Additional file 1: Fig. 1. The overall survival (OS) values were analyzed in regards to the mRNA expression level of CDK19 in differential subgroups of HCC patients. [file 12876_2021_1962_MOESM1_ESM.pdf]

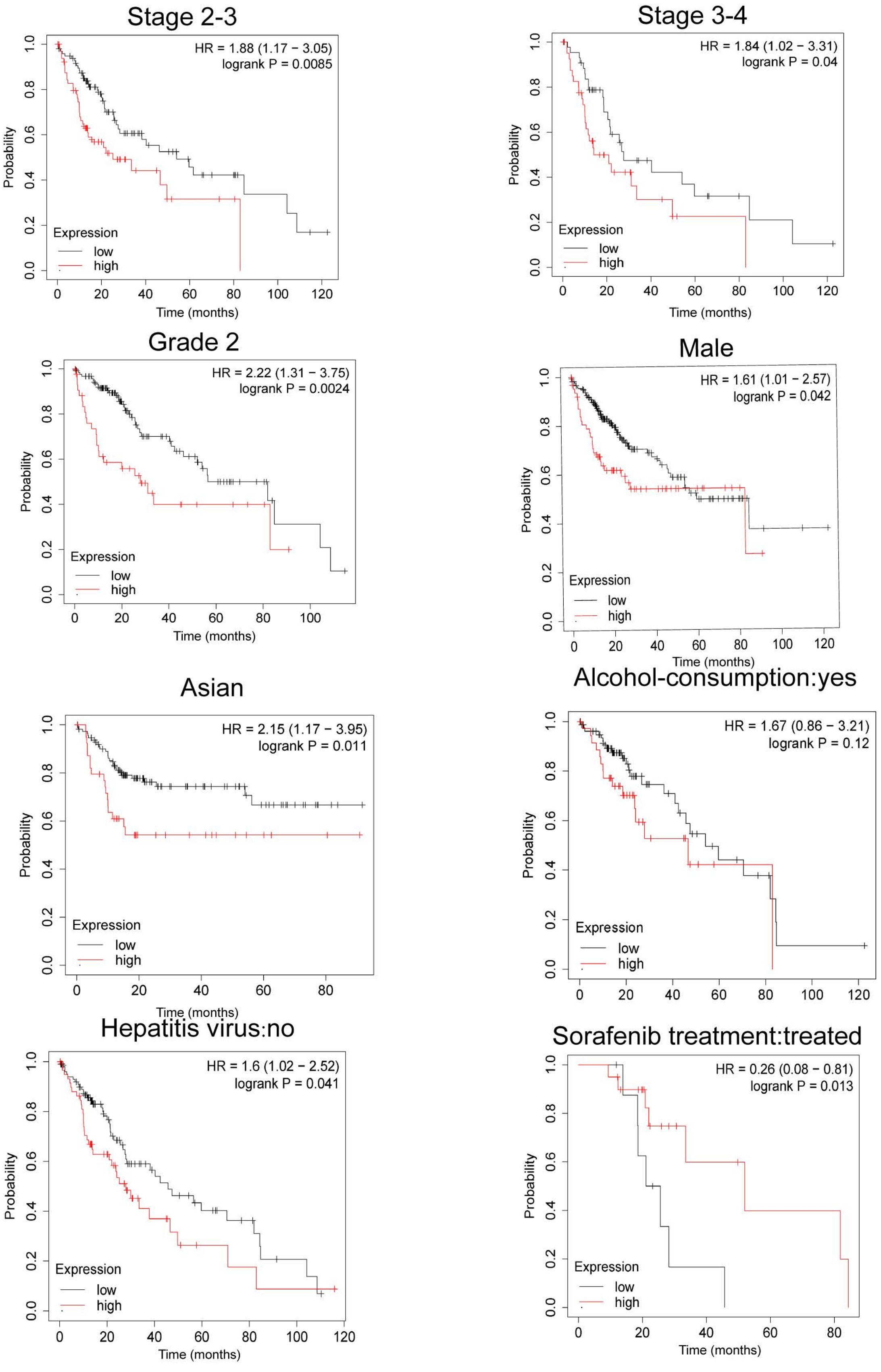
Stage 2-3
HR = 1.88 (1.17 − 3.05)
logrank P = 0.0085
Stage 3-4
HR = 1.84 (1.02 − 3.31)
logrank P = 0.04
Grade 2
HR = 2.22 (1.31 − 3.75)
logrank P = 0.0024
Male
HR = 1.61 (1.01 − 2.57)
logrank P = 0.042
Asian
HR = 2.15 (1.17 − 3.95)
logrank P = 0.011
Alcohol-consumption:yes
HR = 1.67 (0.86 − 3.21)
logrank P = 0.12
Hepatitis virus:no
HR = 1.6 (1.02 − 2.52)
logrank P = 0.041
Sorafenib treatment:treated
HR = 0.26 (0.08 − 0.81)
logrank P = 0.013
Probability
Time (months)
Expression
low
high
