# Supplementary figures and images for: Cyclin-dependent kinase 19 upregulation correlates with an unfavorable prognosis in hepatocellular carcinoma
Source: BMC Gastroenterol. 2021 Oct 14;21:377. doi: 10.1186/s12876-021-01962-8 (PMC8518165; doi:10.1186/s12876-021-01962-8)

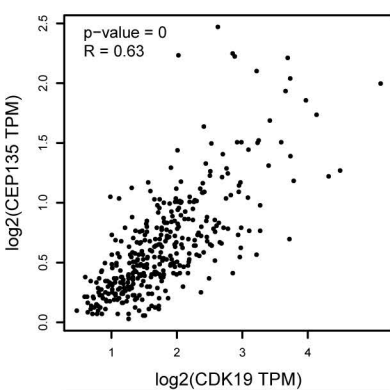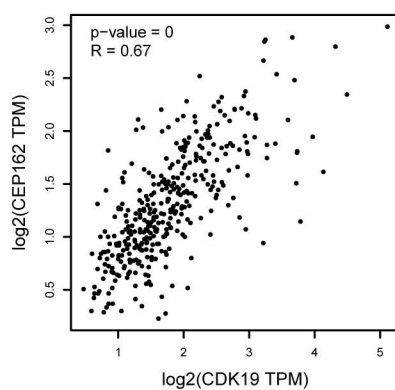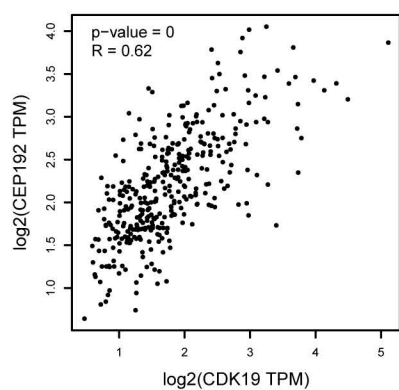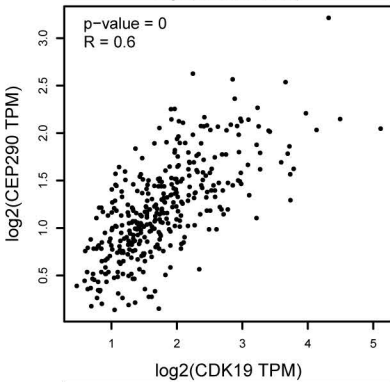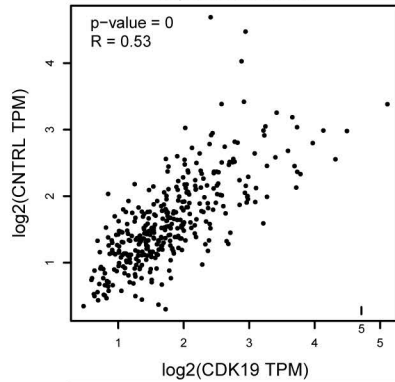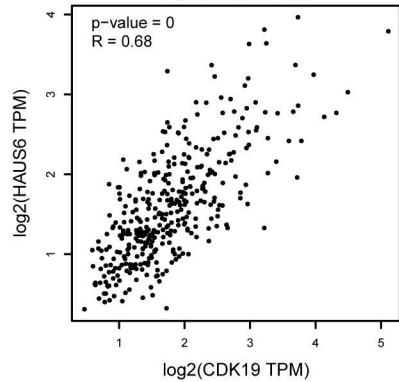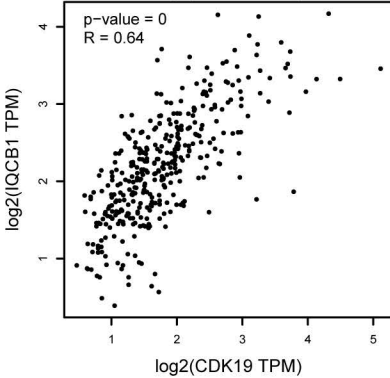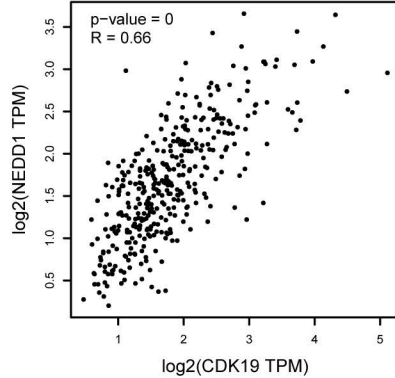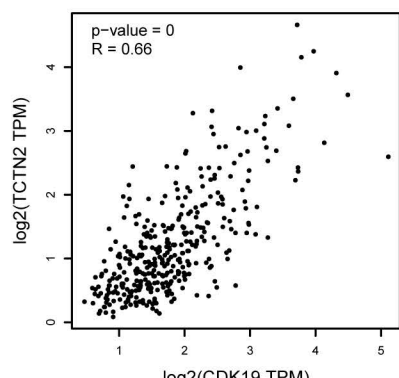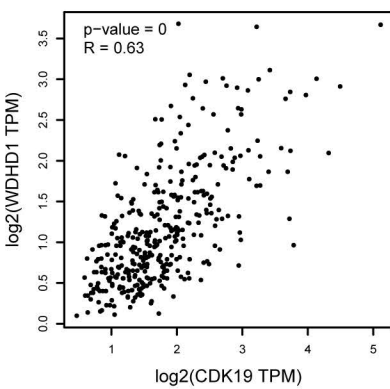

Supplement: Supplementary file 2 — Additional file 2: Fig. 2. The relevance of CDK19 gene expression in relation to the top 10 hub genes. [file 12876_2021_1962_MOESM2_ESM.pdf]

CEP135 (9662)

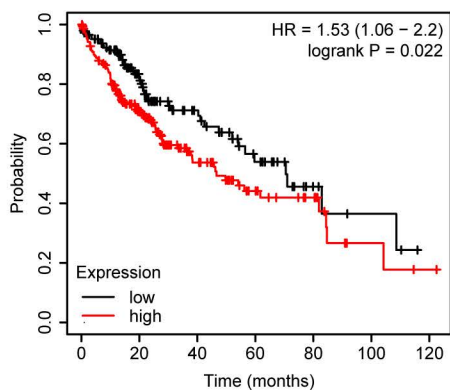

CEP192 (55125)

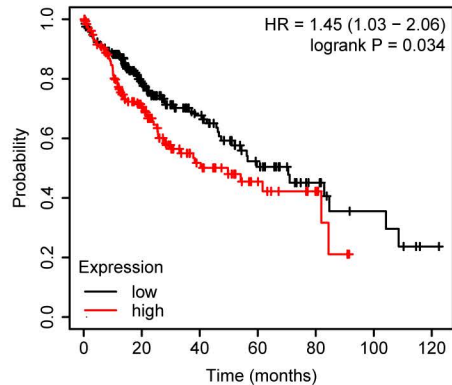

CEP290 (80184)

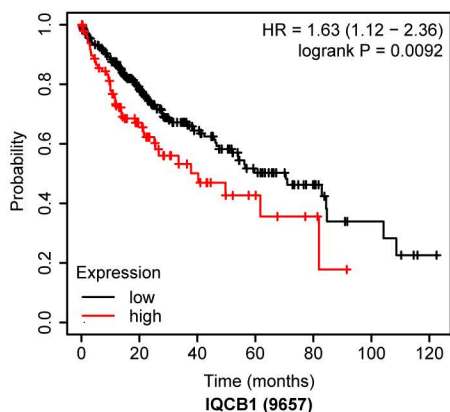

HAUS6 (54801)

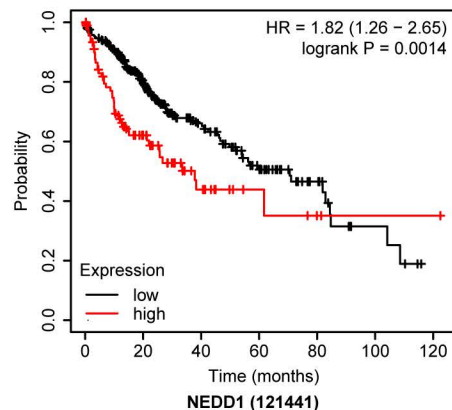

IQCB1 (9657)

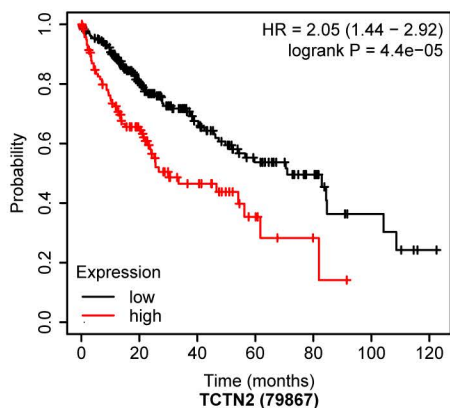

NEDD1 (121441)

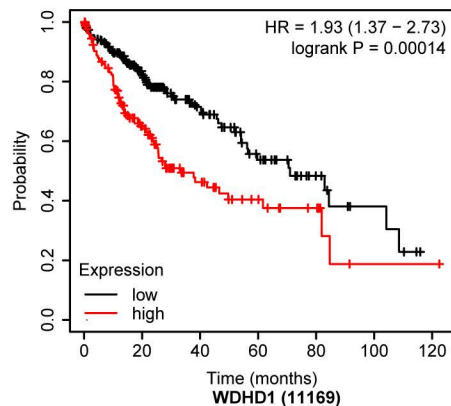

TCTN2 (79867)

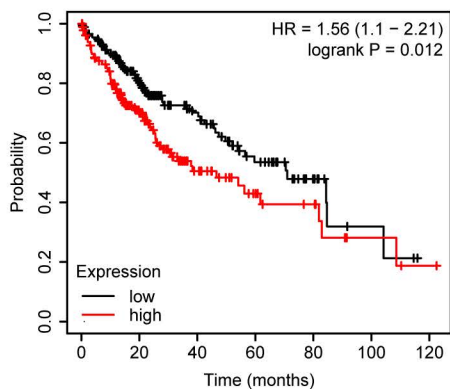

WDHD1 (11169)

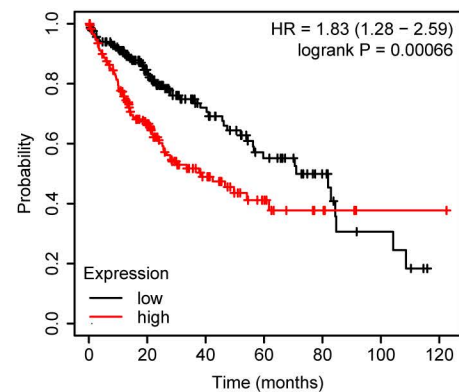

Supplement: Supplementary file 3 — Additional file 3: Fig. 3. The prognostic significance of the top 10 hub genes. [file 12876_2021_1962_MOESM3_ESM.pdf]
